# Biographical Notice of the Late Dr. Gregory
*We have received this notice from an authentic source. The previous arrangements of the Journal had left us so little space when we received it, that we have been obliged to omit several parts of the original narration, but we believe we have preserved whatever is of most importance to the character of the subject of it as a physician, professor, and man of science. The expressions of regret fo his loss and just eulogy of his talents, with which the biography was preceded, will too readily occur to every reader to require that they should be expressed by the historian. *Editor*.


**Published:** 1821-05

**Authors:** 


### BIOGRAPHICAL NOTICE OF THE LATE DR. GREGORY.*

DR. JAMES GREGORY was descended from an ancient family in Aberdeenshire, which for above two centuries has enjoyed a distinguished name in the annals of science, and been particularly eminent for mathematical genius. It is a remarkable fact, that a great number of the descendants of this family have filled with great repute professional chairs in different Universities.

Dr. Gregory's father was professor of medicine, first in Aberdeen and afterwards in Edinburgh. His grandfather was professor of medicine in Aberdeen. His great grandfather, the celebrated cotemporary of Newton, and inventor of the Gregorian Telescope, was professor of mathematics, first at St. Andrew's and afterwards at Edinburgh. The other branches of the family equally contributed to support, in this respect, the honour of the name. The elder, (being that of the Gregories of Kinardy,) even boast of a much larger number. Among them may be noticed Dr. David Gregory, professor of mathematics at Edinburgh and Oxford, the father of Dr. David Gregory, formerly Dean of Christ-Church. Mr. Innes, professor of Philosophy, in Aberdeen; Mr. Charles Gregory, professor of Mathematics, at St. Andrew's. His son, Mr. David Gregory, who succeeded him in the same chair: and lastly, the celebrated Dr. Reid, professor of Logic, at Glasgow.

"Dr. John Gregory, well known to the medical profession by his admirable "*Lectures on the duties and qualifications of a Physician*," and to the world in general as the author of "*A Father's Legacy to his Daughters*," left three sons and two daughters, by Elizabeth, daughter of William Lord Forbes, a lady who, to great beauty and engaging manners, joined a very superior understanding, and a more than common share of wit. The eldest son, James, (the subject of the present memoir,) was born at Aberdeen in January, 1753, and received the rudiments of his education at the grammar-school of his native place. During the winter 1764-5, he studied at King's College, Aberdeen; from that University he was removed next season to Edinburgh, and the following year was entered at Christ

---

* We have received this notice from an authentic source. The previous arrangements of the Journal had left us so little space when we received it, that we have been obliged to omit several parts of the original narration, but we believe we have preserved whatever is of most importance to the character of the subject of it as a physician, professor, and man of science. The expressions of regret fo· his loss and just eulogy of his talents, with which the biography was preceded, will too readily occur to every reader to require that they should be expressed by the historian. *Editor.*

Church, Oxford. He resumed his studies at Edinburgh towards the close of 1767, and continued there until the winter 1773, when he was sent to London to prosecute his medical education. Here he became a pupil of St. George's Hospital. In June, 1774, he graduated at Edinburgh. His thesis is entitled, "*De Morbis Cœli Mutatione Medendis.*" It treats in detail of phthisis, pulmonalis, hypochondriasis, and gout; and concludes by noticing the advantage of change of air in the prolonging of human life. It is marked by the same elegance of composition for which he soon after became so conspicuous. As a medical work, it is chiefly interesting, as containing a very clear exposition of the doctrine of *irregular distributions of blood.*

During the whole of the year 1775 he was on the Continent, and in the course of his tour visited Holland, France, and Italy. Many of his letters written at that time are still extant, and prove that he was feelingly alive to all the beauties of nature, and a fervent admirer of the treasures of antient art.

In June, 1776, he was appointed (at the early age of twenty-three,) to the professorship of the Theory of Physic; and he continued to teach this class with great distinction for twelve years. As a text-book for his lectures he published in 1779-80, the first part of his "*Conspectus Medicinæ Theoreticæ.*" The work was completed in two large octavo volumes in 1782. It rapidly acquired a high reputation all over Europe, not only in consequence of the scientific merits which it possessed, but the singular felicity of classical language in which it was written. Several editions of it have since been published, but it is greatly to be regretted that it never received from the author those additions and alterations which were required to adapt it to the present state of physiological, and more particularly chemical science. Hence its value is now considerably diminished to the student of medicine, but it is still perused with the highest advantage for the general principles of the science, and as a model of classical latinity it will probably remain unrivalled.

In the year 1790 Dr. Gregory was appointed, in consequence of the death of Dr. Cullen, to the chair of the practice of medicine; and for thirty years he sustained, and even increased the celebrity which the eminence of his predecessor had conferred upon the office. His lectures, besides their intrinsic merit as the vehicles of sound medical learning, were characterized by a richness of illustration which we imagine to be quite unprecedented. From very early infancy Dr. Gregory had been remarkable for a most retentive memory, and this he had diligently cultivated. The advantages which it gave him as a teacher of medicine are incalculable. He has been frequently heard to describe cases which occurred in the earliest years of his practice with a freshness, and *particularity* of detail, which made them appear like the observation of the day. No case ever seems to have escaped his memory which could enforce some principle of pathology or practice; and he could draw therefore, almost *ad libitum*, upon these stores, for the most striking illustrations of the doctrine he was inculcating.

It is much to be regretted that he never carried into execution the design which he had long contemplated of publishing a *Conspectus Medicinæ Practicæ.* The mass of medical information which he possessed was immense, and we much fear it has perished with him. No notes of his lectures can, we imagine, ever be considered as giving a correct view of his opinions or practice. We are led to this conclusion by considering, how remarkably *precise* he was in the choice of his expressions, how frequently he varied his lectures from the mere extent of the subjects before him, and with what candour he modified his views according to the experience by which he was continually profiting.

As a literary man, Dr. Gregory long enjoyed a high reputation. He was early devoted to the study of metaphysics, and in combating the doctrine of necessity he displayed uncommon ingenuity. His "*Literary and Philosophical Essays,*" in two vols. were published in 1792. As a philologist, he is known by a very profound essay on "*the Theory of the Moods of Verbs,*" published in the second vol. of the Transactions of the Royal Society of Edinburgh. Though not a professed mathematician, his mind was deeply imbued with the spirit of mathematical science, inherited from his celebrated ancestor; and this can be traced in all his writings, conspicuous alike for closeness of reasoning, clearness of arrangement, and the utmost precision of language.

His controversial writings are numerous, and display an exuberance of talent, a lively wit, and an extent of general information, which is astonishing, when we consider with what assiduity he cultivated, from a very early period of life, his professional pursuits.

In 1781 he married Miss Ross, daughter of James Ross, esq. of Stranraer. By this lady, who did not long survive her marriage, he had not any children. In 1796 he married Miss M'Leod, daughter of Donald M'Leod, esq. of Glanies, in Ross-shire. By her he has had eleven children, of whom seven are living; five sons and two daughters. The eldest son is practising at the Scotch bar; and the second, who bears his father's name, is now qualifying himself to follow that profession, of which his father was so bright an ornament.

To great merits as a teacher of medicine, and high reputation as a man of science and literature, Dr. Gregory added a distinguished character as a *practical* physician. From the time he was first appointed to a chair in the University, he took an active share in the clinical practice of the infirmary; and the lectures by which he illustrated it are spoken of by those who heard them, in terms of unbounded admiration. About the period of his second marriage he began to be actively engaged in private practice. This increased so much upon him, that in less than ten years afterwards he was obliged to givo up his clinical lectures, to the great regret of the students.

In gardening, Dr. Gregory early found a great source of amusement, and he continued to indulge it to within a few days of his decease. At one period of his life he was a zealous archer, at another he entered with spirit upon the duties of a soldier, and never ceased to look back with pleasure upon the time he carried a musket as a private grenadier. Possessing a fine relish for ancient poetry, he occasionally attempted to infuse its spirit into our own language. He was more happy in his Inscriptions, many of which are worthy of the proudest days of Roman literature.

In spring, 1818, he experienced a severe misfortune, in the breaking of his arm, by the accidental oversetting of the carriage in which he was travelling. The injury which he received was very severe, and his health soon after began sensibly to decline. In April, 1820, he visited London, after an absence of twenty-eight years, as one of a deputation from the University of Edinburgh, to congratulate his Majesty on his accession to the throne. On that occasion he was honoured by a private audience of his Majesty; of whose gracious condescension he cherished the most grateful remembrance. During the last year, he suffered severely from attacks of difficulty of breathing, which ultimately ended in hydrothorax, which put a period to his valuable life, soon after having completed his 68th year. His health did not permit him to give any Lectures, during the last winter, after Christmas. The course was completed by his nephew, Dr. W. P. Alison, in a manner which, we hear, has gained the approbation of a very numerous class.

His remains were interred in the family-vault in the Cannongate church-yard, with great solemnity, on Saturday, April 7th; the funeral being attended by the magistrates, professors, and other public bodies, the students of his class, and many private friends. In the Cannongate, where Dr. Gregory at one time resided, a very strong testimony of respect was paid to his memory, by shutting up the shops during the time the procession passed. An universal feeling of regret has indeed been expressed for the loss of one whose life was at once so honourable and so useful. Uniting, in a degree seldom equalled, the studies and acquirements of a man of science with the taste and honourable feelings of a gentleman, he shed a lustre upon the medical profession, whose influence will, we trust, be as lasting as it has been extensive and important.

The loss of such a man will be felt as occasioning a blank, almost irreparable, not only in the academical celebrity of Edinburgh, but even in the national distinction of the country.

